# Therapieentscheidungen beim hepatozellulären Karzinom: Erkenntnisse aus der TRENDY-Studie

**DOI:** 10.1007/s00066-023-02140-3

**Published:** 2023-08-31

**Authors:** Danny Jazmati, Judit Boda-Heggemann, Oliver Blanck, David Krug

**Affiliations:** 1 Klinik für Radioonkologie und Strahlentherapie, Heinrich-Heine-Universität Düsseldorf, Universitätsklinikum Düsseldorf, Düsseldorf, Deutschland; 2 Klinik für Strahlentherapie und Radioonkologie, Universitätsmedizin Mannheim, Medizinische Fakultät Mannheim, Universität Heidelberg, Mannheim, Deutschland; 3 Klinik für Strahlentherapie, Universitätsklinikum Schleswig-Holstein, Campus Kiel, Kiel, Deutschland; 4 Arbeitsgruppe junge DEGRO der Deutschen Gesellschaft für Radioonkologie e. V., Berlin, Deutschland

## Hintergrund der Arbeit

Patient:innen mit einem hepatozellulären Karzinom (HCC) stehen mehrere potenziell kurative Behandlungsoptionen zur Verfügung, darunter Lebertransplantation, Leberresektion und Radiofrequenzablation (RFA; [6]). Bei Betroffenen, die für einen chirurgischen Eingriff oder eine RFA nicht infrage kommen oder diese ablehnen, stellt die Wahl der optimalen Behandlung eine große Herausforderung dar. Die transarterielle Chemoembolisation (TACE) ist zwar als lokale Behandlungsoption für inoperable HCC etabliert, doch die Verbesserung der onkologischen Endpunkte im Vergleich zu einer rein supportiven Therapie bleibt moderat [1]. Vielversprechende Ergebnisse konnten mit der stereotaktischen Strahlentherapie (SBRT) bei inoperablem oder rezidivierendem HCC erreicht werden [2, 7, 8]. Nichtsdestotrotz gibt es nur wenige vergleichende Studien zwischen SBRT und TACE, was eine Abwägung der Chancen und Risiken für den individuellen Patienten limitiert. Aus diesem Grund ist ein direkter Vergleich dieser beiden Behandlungsmodalitäten wünschenswert und wurde von den Autoren der vorliegenden Studie adressiert.

## Studiendesign/Methode

Diese multizentrische prospektive Phase-II-Studie vergleicht randomisiert die onkologische Wertigkeit zwischen der transarteriellen Chemoembolisation mit „drug-eluting beads“ (TACE-DEB) und der stereotaktischen Strahlentherapie (SBRT) bei Patient:innen mit HCC, die für eine Operation oder Ablation nicht infrage kamen. Einschlusskriterien waren ein bildgebend gesichertes HCC mit ein bis drei Läsionen und einem kumulativen Durchmesser von ≤ 6 cm, wobei maximal eine Leberzirrhose im Stadium Child-Pugh A bestehen durfte. Bei der TACE-DEB wurden mit Doxorubicin beladene Mikrosphären verabreicht, wobei eine Wiederholung der Applikation möglich war (3 von insgesamt 16 Patient:innen). Bei der SBRT wurde, entsprechend dem kanadischen Protokoll, eine risikoangepasste Dosis von 6 × 8–9 Gy verschrieben. Primärer Endpunkt war die Zeit bis zur Progression (TTP). Gezeigt werden sollte eine Verbesserung der TTP von 16 Monate mit DEB-TACE auf 36,5 Monate mit SBRT (Hazard Ratio [HR] 0,438). Sekundäre Endpunkte waren die lokale Kontrolle, das Gesamtüberleben, die Ansprechrate, die Toxizität und die Lebensqualität. Die Studie sah vor, dass 100 Patienten rekrutiert werden sollten, welche gleichmäßig auf die beiden Behandlungsarme verteilt wurden. Leider wurde die Studie wegen unzureichender Rekrutierung frühzeitig geschlossen.

## Ergebnisse

Zwischen dem 1. Mai 2015 und dem 14. April 2020 wurden insgesamt 30 Patienten in die Studie eingeschlossen, von denen 28 in die Auswertung eingingen. Die mediane Nachbeobachtungszeit betrug 28,1 Monate. Die Ansprechraten betrugen 81 % für TACE-DEB und 92 % für SBRT. Es gab einen numerischen Vorteil der SBRT hinsichtlich der TTP (18,8 vs. 12,0 Monate; HR 0,45; *p* = 0,15). Darüber hinaus betrug die mediane Zeit bis zum Lokalrezidiv für TACE-DEB 12 Monate, während sie für SBRT nicht erreicht wurde (> 40 Monate; HR 0,15; *p* = 0,075). Für beide Endpunkte zeigte sich jedoch keine statistische Signifikanz.

In einer Post-hoc-Analyse, bei der Patienten, die in der SBRT-Gruppe nicht gemäß dem Protokoll behandelt wurden, ausgeschlossen wurden, zeigte sich mit einer LC-Rate von 100 % eine signifikante Überlegenheit der SBRT (stratifizierter Log-Rang-Test, *p* = 0,019). Bei der TACE-DEB wurden drei Fälle von behandlungsbedingter Grad-3-Toxizität berichtet, während bei der SBRT keine Grad-3-Toxizität festgestellt wurde. Die Lebensqualität blieb in beiden Gruppen stabil. Hinsichtlich des Gesamtüberlebens fand sich kein signifikanter Unterschied.

## Schlussfolgerung der Autoren

Die SBRT zeigte im Vergleich zur TACE-DEB eine höhere lokale antitumorale Aktivität ohne Nachteile im Hinblick auf Toxizität, Lebensqualität oder Überleben. Die Autoren betonen, dass internationale und interdisziplinäre Kollaborationen wünschenswert sind, um die Herausforderung der begrenzten Patientenzahl zu bewältigen.

## Kommentar

Die Strahlentherapie hat in der Behandlung des HCC einen schweren Stand. Trotz vielversprechender Ergebnisse der SBRT in nahezu jedem Therapiesetting mit lokalen Kontrollraten von ca. 90 % und einer höheren Radiosensibilität des HCC gegenüber Lebermetastasen wird die SBRT in den großen internationalen Leitlinien stiefmütterlich behandelt. Eingeschränkt ist die Datenlage durch die zumeist kleinen Studien, die häufig aus dem asiatischen Raum kommen und aufgrund der unterschiedlichen Genese der fast immer zugrunde liegenden Leberzirrhose nur bedingt übertragbar sind. In der deutschen S3-Leitlinie wird die SBRT bislang nur als Option betrachtet, wenn andere Therapieverfahren nicht oder nur eingeschränkt zum Einsatz kommen können. Als Beispiele zu nennen sind Tumoren > 3 cm, ein direkter Gefäßbezug (jeweils assoziiert mit abnehmender Effektivität der radiologisch-interventionellen Verfahren), eine ungünstige Lage für alternative Verfahren (z. B. Leberkuppel oder angrenzend ans Gallenblasenbett) und ein eingeschränkter Gefäßstatus (für TACE).

Während Resektion und RFA bzw. Mikrowellenablation (MWA) als kurative Verfahren betrachtet werden, ist die TACE kein lokal ablatives, sondern ein palliatives Therapieverfahren. Typischerweise wird die TACE repetitiv angewendet.

Bislang fehlte es an hochqualitativen vergleichenden Studien zwischen SBRT und TACE. In einer ebenfalls kleinen randomisierten Studie untersuchten Comito et al. kürzlich die SBRT nach einem Zyklus TA(C)E mit einer fortgesetzten TA(C)E bei Patienten mit inkomplettem Ansprechen nach dem ersten TA(C)E-Zyklus. Es wurden 41 Patienten eingeschlossen [4]. Die lokale Tumorkontrolle (Median nicht erreicht vs. 8 Monate; HR 0,15; *p* = 0,0002) und das progressionsfreie Überleben (Median 9 vs. 4 Monate; HR 0,43; *p* = 0,016) wurden durch die SBRT signifikant verbessert. Gesamtüberleben und Toxizität waren vergleichbar. In dieser Studie erhielten jedoch nur 21 % der Patienten eine tatsächliche TACE während der Rest der Patienten eine Embolisation ohne Chemotherapiebeladung bekam.

In der deutschen HERACLES-Studie, einer prospektiven Beobachtungsstudie, wurden ebenfalls TACE und SBRT verglichen. Insgesamt erhielten 19 Patienten eine TACE und 18 Patienten eine SBRT [3]. Das Risiko einer lokalen Tumorprogression betrug nach 3 Jahren 6 % nach SBRT vs. 65 % nach TACE (*p* = 0,02). Das mediane progressionsfreie Überleben war jedoch nach TACE mit 11 Monaten länger als nach SBRT mit 4 Monaten (*p* = 0,05). Aufgrund der fehlenden Randomisierung und der fortgeschritteneren Stadien in der SBRT-Gruppe ist dies jedoch nur eingeschränkt aussagekräftig.

In einer retrospektiven Studie wurden SBRT und TACE bei 209 Patient:innen nach „propensity score matching“ verglichen. Die 1‑ und 2‑Jahres-LC-Raten zeigten eine signifikante Überlegenheit der SBRT-Gruppe im Vergleich zur TACE-Gruppe: 97 % bzw. 91 % für SBRT und 47 % bzw. 23 % für TACE (Hazard Ratio 66,5, *p* < 0,001). Die Rate an Grad-≥ 3-Toxizität betrug 13 % für TACE und 8 % für SBRT-Behandlungen (*p* = 0,05; [7]).

Bei fehlenden gesicherten prospektiven Daten deuten diese beiden Studien auf einen potenziellen Synergieeffekt hin, wenn die SBRT in multimodale Behandlungsstrategien integriert ist. Dies verdeutlicht jedoch auch die Komplexität der Entscheidungsfindung und unterstreicht die Notwendigkeit für eine weitere wissenschaftliche Auseinandersetzung.

Auch zur Therapie im fortgeschrittenen Stadium mit Indikation zur palliativen Systemtherapie gibt es Daten zur Integration einer SBRT. In einer retrospektiven multizentrischen Studie verglichen Bettinger et al. insgesamt 901 mit Sorafenib und 122 mit SBRT (ohne Systemtherapie) behandelte Patient:innen mit fortgeschrittenem HCC [2]. Das mediane Gesamtüberleben der SBRT-Patienten betrug im Vergleich zu den Sorafenibpatienten 18,1 Monate gegenüber 8,8 Monaten [2]. Auch die kürzlich präsentierte RTOG-1112-Studie untersuchte wiederum die Hinzunahme der SBRT zur Systemtherapie mit Sorafenib bei fortgeschrittenem HCC. Die kombinierte Therapie mit SBRT und Sorafenib führte im Vergleich zu einer alleinigen Sorafenibtherapie zu einer Verbesserung des medianen OS von 12,3 auf 15,8 Monate (HR = 0,77) und des medianen PFS von 5,5 auf 9,2 Monate (HR = 0,55; *p* = 0,001; [5]). Aufgrund der sich schnell ändernden Systemtherapielandschaft wird die Studie allerdings kritisch diskutiert, da Sorafenib nicht mehr die empfohlene Erstlinientherapie ist. Aktuelle Studien untersuchen, ob die zusätzliche SBRT auch bei einer Therapie mit Atezolizumab/Bevacizumab einen „benefit“ erbringt (NCT05488522). Nichtsdestotrotz ist diese Kombinationstherapie mit Sorafenib eine Option für Patienten, die keine Immuntherapie erhalten können – z. B. wegen einer vorangegangenen Lebertransplantation.

Ein kritischer Aspekt der hier vorgestellten TRENDY-Studie ist die geringe Patientenzahl, die zu einer Einschränkung der statistischen Aussagekraft der Ergebnisse führt. Von den ursprünglich geplanten 100 Patient:innen konnten letztendlich nur 28 eingeschlossen werden. Die geringe Anzahl der Rekrutierungen für die vorliegende Studie lässt sich durch eine Vielzahl von Faktoren erklären. In ganz Europa, einschließlich der Niederlande, ist die Inzidenz des HCC insgesamt niedrig. In Deutschland wurde die Studie in mehreren Zentren initiiert, jedoch konnten keine Patienten eingeschlossen werden, was zumindest zum Teil auf die Einschlusskriterien zurückzuführen ist. Diese Kriterien beschränkten sich auf eine Leberzirrhose mit geringer Einschränkung der Leberfunktion (Child-Pugh-Stadium A) mit einem maximalen kumulativen Tumordurchmesser von 6 cm und schlossen gleichzeitig Patienten mit makrovaskulärer Infiltration, Aszites und ösophagealen Varizen aus.

Ein weiterer wichtiger Aspekt, der aus dieser Studie hervorgeht, ist die Bedeutung der radioonkologischen Qualität, um optimale Behandlungsergebnisse zu erzielen. Die Definition und Anwendung von Qualitätskriterien für die Strahlentherapie beim HCC sind nur unzureichend definiert. Größere prospektive Studien oder Registerstudien sind notwendig, um detailliertere Qualitätskriterien zu etablieren und eine optimale Strahlentherapie für Patient:innen mit HCC ermöglichen zu können. Aufgrund der historisch heterogenen Daten hat sich bisweilen kein Standardtherapiekonzept etabliert. Die hohe Ansprechrate und die verbesserte lokale Kontrolle, die mit der Strahlentherapie im Vergleich zur TACE beobachtet wurden, zeigen, dass die SBRT eine vielversprechende und effektive Therapieoption für Patient:innen mit HCC darstellt.

Das deutsche HepReg-Register soll ab 08/2023 Patient:innen mit SBRT für ein HCC retrospektiv und prospektiv multizentrisch einschließen und bietet das Potenzial, auf Basis einer großen Datenmenge Rückschlüsse zur optimalen Strahlentherapie für zukünftige prospektive randomisierte Studien und Leitlinienempfehlungen ziehen zu können.

*Danny Jazmati, Judit Boda-Heggemann, Oliver Blanck, David Krug*
